# Risk factors and treatments for disseminated intravascular coagulation in neonates

**DOI:** 10.1186/s13052-020-0815-7

**Published:** 2020-04-29

**Authors:** Hayato Go, Hitoshi Ohto, Kenneth E. Nollet, Nozomi Kashiwabara, Kei Ogasawara, Mina Chishiki, Shun Hiruta, Ichiri Sakuma, Yukihiko Kawasaki, Mitsuaki Hosoya

**Affiliations:** 1 Department of Pediatrics, Fukushima Medical University School of Medicine, Fukushima, 960-1295 Japan; 2 Department of Blood Transfusion and Transplantation Immunology, Fukushima Medical University School of Medicine, Fukushima, Japan

**Keywords:** DIC score, Underlying conditions, Fresh frozen plasma, Recombinant thrombomodulin, Neonates, Birth asphyxia

## Abstract

**Background:**

Although disseminated intravascular coagulation (DIC) is a critical disease, there is few gold standard interventions in neonatal medicine. The aim of this study is to reveal factors affecting neonatal DIC at birth and to assess the effectiveness of rTM and FFP for DIC in neonates at birth.

**Methods:**

We retrospectively evaluated DIC score on the first day of life in neonates with underlying conditions associated with DIC. DIC in neonates was diagnosed according to Japan Society of Obstetrical, Gynecological & Neonatal Hematology 2016 neonatal DIC criteria.

**Results:**

Comparing neonates with DIC scores of ≥3 (*n* = 103) to those < 3 (*n* = 263), SGA, birth asphyxia, low Apgar score, hemangioma, hydrops, PIH, and PA were statistically increased. Among 55 neonates underwent DIC treatment, 53 had birth asphyxia and 12 had intraventricular hemorrhage. Forty-one neonates received FFP or a combination of FFP and antithrombin (FFP group), while 14 neonates received rTM or a combination of rTM, FFP, and antithrombin (rTM group). DIC score before treatment in the rTM group was significantly higher than in the FFP group (4.7 vs 3.6, *P* < 0.05). After treatment, DIC scores in both groups were significantly reduced on Day 1 and Day 2 (P < 0.05).

**Conclusions:**

Among various factors associated with DIC in neonates at birth, birth asphyxia is particularly significant. Furthermore, rTM in combination with FFP therapy was effective for neonatal DIC at birth.

## Introduction

Although disseminated intravascular coagulation (DIC) is a critical disease [1–3], there is few gold standard interventions in neonatal medicine. Veldman et al. suggested that DIC in neonates is caused by prenatal risk factors, such as placental abruption (PA), pregnancy-induced hypertension (PIH), and neonatal factors such as sepsis, asphyxia and intravascular hemorrhage (IVH) [4]. Especially at birth, asphyxia was noteworthy in these cases. We previously reported that coagulation parameters, such as prothrombin time-international normalized ratio (PT-INR), activated prothrombin time (aPTT), and D-dimer are all affected by PA, birth asphyxia and IVH. Anticoagulant therapy, such as antithrombin administration and fresh frozen plasma (FFP), has been used to treat neonatal DIC. Since 2008, recombinant human soluble thrombomodulin (rTM) has emerged as a novel anticoagulant for DIC in Japan. Previous studies have reported rTM to be effective for DIC in both pediatric and adult patients [5, 6]. In adults, the efficacy of combined rTM and antithrombin therapy for sepsis-associated DIC has been reported [6]. However, there are few reports about the effects of rTM on premature infants. The Japan Society of Obstetrical, Gynecological & Neonatal Hematology (JSOGNH) recommends rTM administration for the treatment of DIC in neonates, and revised its diagnostic guidelines for neonatal DIC in 2016 [7]. We also reported gestational age (GA) and birth weight (BW) to be related to coagulation parameters [8]. Therefore, it is important to measure coagulation parameters in ill neonates with perinatal risk factors for DIC at birth and diagnose DIC in neonates. The aims of this study were to investigate the underlying conditions affecting DIC at birth and to assess the effectiveness of rTM and FFP therapy.

## Methods

### Study design and population

This retrospective cohort study, using records from January 2010 to December 2017, was conducted at the Neonatal Intensive Care Unit (NICU) of Fukushima Medical University Hospital (FMU). We enrolled only neonates born at FMU, but excluded those who were transferred to NICU. The Ethics Committee of FMU, guided by local policy, national law, and the World Medical Association Declaration of Helsinki, approved this study without requiring informed consent from guardians.

### Coagulation factor measurement

Samples for coagulation testing, routinely drawn on the first day of life, usually within 12 h of birth, were non-heparinized peripheral venous or peripheral arterial blood as previously described [8]. Coagulation tests were performed using a Sysmex CS-5100 coagulation analyzer (Sysmex, Kobe, Japan).

### Diagnosis and treatment of DIC

**Fig. 1:**
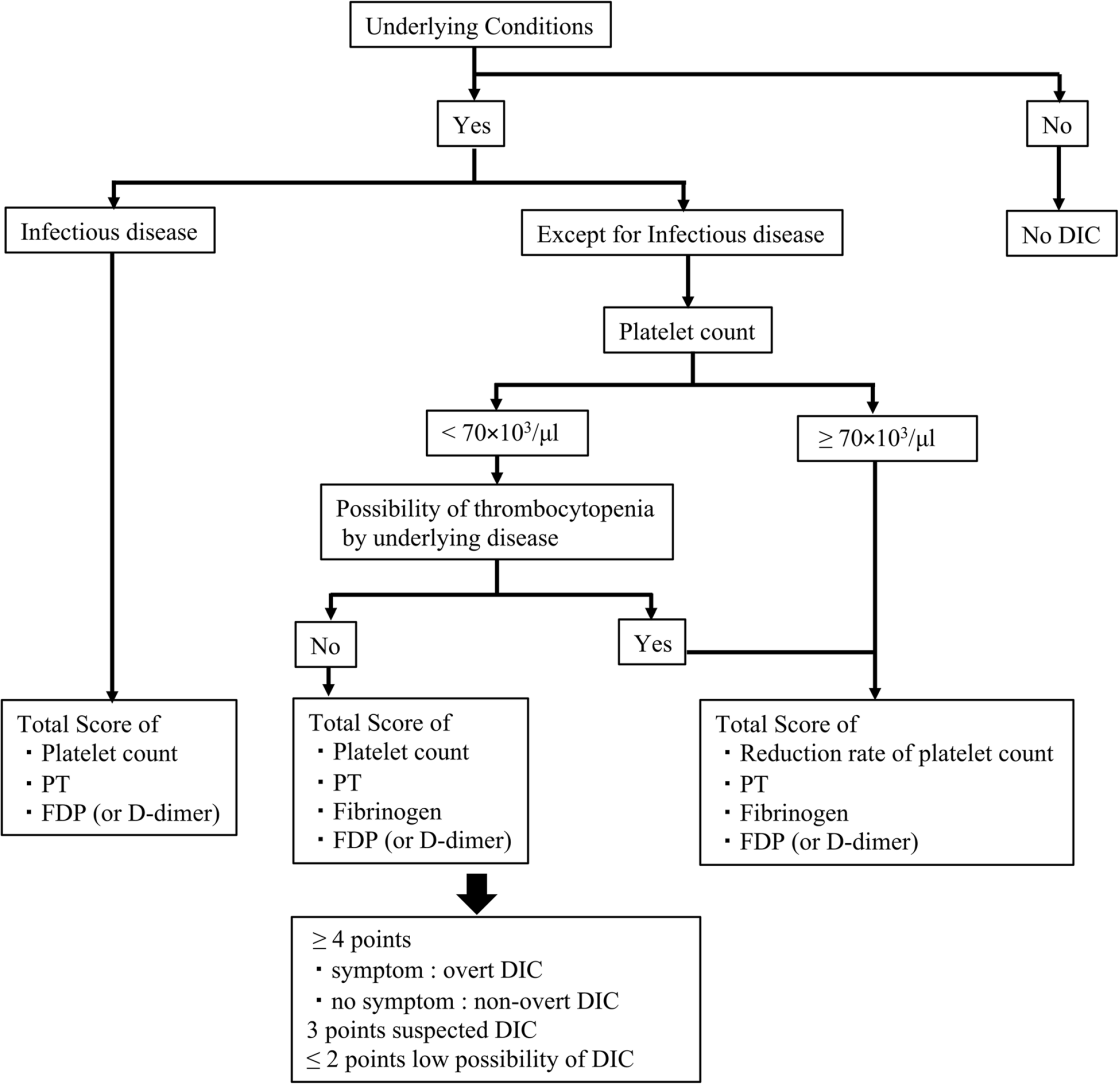
Neonatal DIC algorithm from JSOGNH, 2016

Neonatal DIC algorithm from JSOGNH at 2016 was shown in Fig. 1. DIC was defined as the presence of coagulopathy, thrombocytopenia, low fibrinogen, and elevated D-dimer or FDP on postnatal screening (Table 1). Anticoagulant therapy, such as antithrombin administration, and fresh frozen plasma (FFP), have been used to treat neonatal DIC (Table 1). Among the underlying conditions of DIC in neonates at birth, we included pregnancy-induced hypertension (PIH) and placental abruption (PA) to be prenatal risk factors [4, 9]. In this study, postnatal risk factors were defined as small for gestational age (SGA), respiratory distress syndrome (RDS), intraventricular hemorrhage (IVH), hemangioma, hydrops, sepsis, and a low Apgar score (Apgar Score < 4 at 5 min) [4, 10].

**Table 1:** DIC diagnostic criteria of JSOGN

BW		≥ 1,500g	< 1,500g
PLT	70×103/μL ≤ and 50% reduction within 24 hours	1 point	1 point
	50×103/μL ≤ < 70×103/μL	1 point	1 point
	< 50×103/μL	2 points	2 points
Fibrinogen	50mg/dL ≤ < 100mg/dL	1 point	-
	< 50mg/dL	2 points	1 point
PT-INR	1.6 ≤ < 1.8	1 point	-
	1.8 ≤	2 points	1 point
FDP or D-dimer	< 2.5 fold upper limit of normal range	-1 point	-1 point
	2.5 fold upper limit of normal range ≤ < 10 fold upper limit of normal range	1 point	1 point
	10 fold upper limit of normal range ≤	2 points	2 points

*JSOGNH* Japan Society of Obstetrical, Gynecological & Neonatal Hematology, *PLT* platelet count, *BW* birth weight, *PT-INR* prothrombin time international ratio

For a platelet count of ≥ 70×103 /μL, a point is added if the platelet count is reduced by 50% within 24 hours. A point is not added if the patients had thrombocytopenia due to myelosuppression disease

For fibrinogen, a point is added if the underlying disease of the patient was an infection

Since the upper limit of D-dimer is different among D-dimer kits, a point is added if FDP and D-dimer increased by 2.5 or 10 fold of the upper limit of normal

During the study period, we used two diagnostic criteria for DIC: the conventional DIC criteria used from Jan 2010 to Dec 2015, compiled by Shirahata et al. [11], and the 2016 neonatal DIC diagnostic criteria of the JSOGNH (Table 1) [7]. In this study, we retrospectively validated DIC score using the JSOGNH algorithm for neonatal DIC diagnosis shown in Fig. 1 [7].

As interventions, FFP (10–20 ml/kg), rTM (380 IU/kg), platelet transfusion (10–20 ml/kg) and antithrombin III (AT) (60 IU/kg) were used to treat neonatal DIC.

### Effects on DIC scores and resolution after DIC treatment

DIC scores were retrospectively analyzed using neonatal DIC diagnostic criteria of JSOGNH (Table 1), in which scores > 3 define neonatal DIC and scores =3 are suspected DIC (Fig. 1). Furthermore, *overt* DIC was defined as having bleeding symptoms, and *non-overt* DIC was defined as having no bleeding symptoms on the basis of the guidelines recommended initiating therapy for neonatal DIC at a score ≥ 4. Neonatologists determined treatments of neonates.

### Statistical analysis

PT-INR, aPTT, FBG, d-dimer, and AT were obtained from medical records, and the data were presented as median interquartile range (IQR) percentiles for continuous variables of non-normal distribution. SPSS for Mac, release 25.0 (SPSS, Chicago, IL) was used to perform the statistical analyses. We regarded *P* < 0.05 as statistically significant.

## Results

Of the 985 neonates admitted to our NICU between January 2010 and December 2017, 609 consecutive neonates born at 22–41 weeks were assessed for coagulation factors within the first 12 h of life, as previously described [8]. Of these, 366 neonates had at least one of underlying conditions and the remaining 243 neonates did not. The coagulation parameters and characteristics of the neonates with and without underlying conditions are shown in Supplementary Table 1. As shown in Supplementary Table 2, among 366 neonates with underlying conditions, 168 neonates were <  1500 g and 198 neonates were ≥ 1500 g, respectively.

Comparing neonates with DIC scores of ≥3 (*n* = 103) to those < 3 (*n* = 263), the median GAs and BW at delivery were smaller (Table 2). IVH, SGA, birth asphyxia, low Apgar score, hemangioma, hydrops, PIH, and PA were statistically more common among neonates with scores ≥3. On the other hand, RDS did not correlate with DIC score. Of the 103 neonates with a score ≥ 3, 33 and 70 neonates were diagnosed with neonatal DIC and suspected DIC at birth, respectively. Among 55 neonates who underwent DIC treatment, 53 (91%) had birth asphyxia and 12 (42%) had IVH (Table 3). The remaining 48 neonates did not receive DIC treatment, because they did not have DIC symptoms and the DIC scores of these neonates were all 3. Eleven cases had bleeding symptoms other than IVH, such as purpura, pulmonary hemorrhage, and bleeding during catheterization. As for prenatal factors, the number of neonates born to mothers with PIH and PA were 18 (30%) and 17 (27%), respectively (Table 3). Among the neonates that underwent DIC treatment, six died by Day 28 (Table 4). As shown in Table 4, the bleeding rate in died neonates was significantly higher than that in survivors. However, there were no significant differences in PT-INR, aPTT, FBG, D-dimer and AT.

**Table 2:** Characteristics of DIC and non-DIC neonates

	DIC score ≥ 3 (n = 103)	DIC score < 3 (n = 263)	P-Value
GA (weeks) (median)	29.4	36.4	< 0.001
BW (g) (median)	1342	1703	< 0.001
IVH (%)	21 (20)	20 (8)	< 0.01
SGA (%)	28 (27)	32 (12)	< 0.01
Apgar Score (1 min) (median)	3	5	< 0.001
Apgar Score (5 min) (median)	6	7	< 0.001
Sepsis	1 (1)	0 (0)	0.109
Birth Asphyxia (%)	95 (92)	213 (80)	< 0.001
Low Apgar Score (%)	27 (26)	39 (14)	< 0.01
RDS (%)	29 (28)	68 (26)	0.65
Hemangioma	2 (2)	0 (0)	< 0.05
Hydrops	6 (6)	4 (2)	< 0.05
PIH	16 (15)	12 (5)	< 0.01
PA	15 (15)	11 (4)	< 0.01
PLT (× 10^3^μl) (median)	163	237	< 0.001
PT-INR (median)	1.60	1.27	< 0.001
aPTT (seconds) (median)	78.4	60.9	< 0.01
FBG (mg/dl) (median)	91	131	< 0.001
D-dimer (ng/ml) (median)	12.8	9.1	< 0.001
AT activity (%) (median)	27.5	36.0	< 0.001

*GA* Gestational age, *IVH* Intraventricular hemorrhage, *BW* birth weight, *PT-INR* Prothrombin time international ratio, *RDS* Respiratory distress syndrome, *PIH* Pregnancy induced hypertension, *PA* Placental abruption, *DIC* Disseminated intravascular coagulation, *NS* not significant

*GA* Gestational age, *BW* Birth weight, *IVH* Intraventricular hemorrhage, *SGA* Small for gestational age, *RDS* respiratory distress syndrome, *PIH* pregnancy-induced hypertension, *PA* Placental abruption, *PT-INR* Prothrombin time international normalized ratio; GA, BW, Apgar score, PLT, PT-INR, aPTT, FBG, D-dimer, and AT were analyzed by Mann-Whitney U test. IVH, SGA, sepsis, birth asphyxia, low Apgar score, RDS, hemangioma, hydrops, Continuous variables were presented as median. PIH and PA (%) were analyzed by χ^2^ test

**Table 3:** Underlying conditions in the DIC treatment group

DIC score	(≥ 4 overt DIC: *n* = 10) (≥ 4 non-overt DIC: *n* = 23) (= 3: *n* = 22)
	N(%)
Birth Asphyxia	53(91%)
IVH	12(22%)
Bleeding other than IVH	11(20%)
RDS	18(33%)
PIH	17(30%)
PA	15(27%)
Hydrops	9(16%)
Hemangioma	2(4%)
Vanishing twin	2(4%)
Sepsis	1(2%)

*IVH* Intraventricular hemorrhage, *RDS* Respiratory distress syndrome, *PIH* Pregnancy induced hypertension, *PA* Placental abruption, *DIC* Disseminated intravascular coagulation

**Table 4:** Differences in coagulation parameters between died and survived neonates in DIC treatment group

	Died (*n* = 6)	Survived (*n* = 49)	*P*-value
PLT (10^3^/μL)	170	150	0.555
PT-INR	2.07	1.89	0.850
aPTT (second)	86.0	78.5	0.988
FBG (mg/dl)	85	85	0.169
D-dimer (ng/ml)	32.0	14.3	0.096
AT activity (%)	29.0	27.5	0.515
Bleeding (%)	4 (66%)	3 (7%)	0.006
DIC score	4	4	0.887

*PLT* Platelet count, *PT-INR* Prothrombin time international ratio, aPTT activated partial thrombin time, *FBG* Fibrinogen, *AT* Activity, antithrombin activity, *DIC* disseminated intravascular coagulation. Continuous variables were presented as medians. PLT, PT-INR, aPTT, FBG, D-dimer, AT and DIC score were analyzed by Mann-Whitney U test. Bleeding (%) was analyzed by χ^2^ test

In terms of DIC treatment, we used FFP transfusion (*n* = 55), rTM infusion (*n* = 4), ATIII infusion (*n* = 38), platelet transfusion (*n* = 17) and blood exchange (*n* = 2) (Supplementary Table 3). Among the neonates treated for DIC, 41 received FFP or a combination of FFP and antithrombin (FFP group), and 14 received rTM or a combination of rTM and FFP and antithrombin (rTM group).

**Fig. 2:**
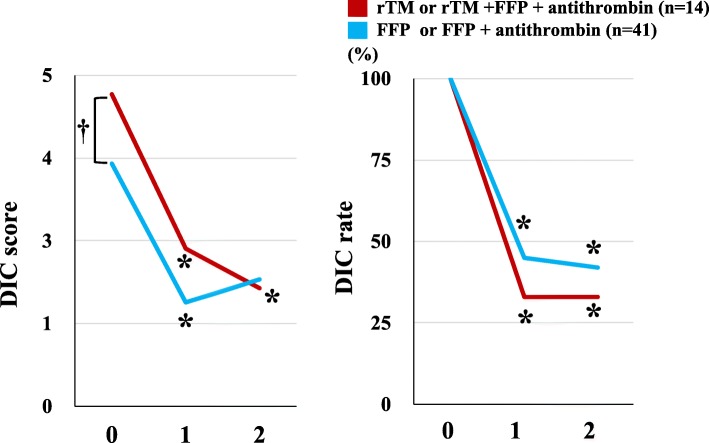
DIC resolution and DIC score after DIC treatment. Data are analyzed by Mann-Whitney U test or χ^2^ test. (A) Y-axis shows DIC score. X-axis shows days after DIC treatment. *p < 0.05 vs. day 0, † *p* < 0.01 DIC score in the rTM group vs. the FFP group. (B) Y-axis shows the rate of DIC diagnosis. X-axis shows day after DIC treatment. *p < 0.05 vs. day 0

DIC score before treatment in the rTM group was significantly higher than in the FFP group (4.7 vs 3.6, *P* < 0.05). After treatment, DIC scores in both groups were significantly reduced on Day 1 and Day 2 (P < 0.05). Furthermore, the rate of DIC was significantly decreased in both groups on days 1 and 2, compared to the rate prior to treatment (Fig. 2).

## Discussion

The present study demonstrated that neonatal DIC at birth is caused by underlying conditions, and that mainly birth asphyxia was the leading risk factor. Previous studies have already suggested that neonates with hypoxic-induced encephalopathy were at risk for hemostatic dysfunction [12, 13]. Previous studies also showed that the prevalence of hemostatic dysfunction with HIE due to perinatal asphyxia was 18–69% [13–16]. Furthermore, in the present study, we observed that prenatal maternal complications such as PA and PIH were found to be risk factors affecting DIC score. These maternal complications are reported to be strongly correlated with birth asphyxia and coagulopathy [17, 18]. Suzuki et al. showed that plasma levels of thrombin-antithrombin complexes, D-dimer, fibrinogen, and fibrin degradation products in cord blood are higher in infants with birth asphyxia [19]. We previously described that maternal and neonatal complications such as birth asphyxia and PA affect coagulation parameters in preterm and term neonates [8]. In adults, most underlying conditions of DIC are reported to be infectious diseases, malignancies, trauma, and brain injury [1, 20, 21], while, we sometimes experience neonatal DIC with sepsis and necrotizing enterocolitis after birth; however, there were few cases of sepsis-associated neonatal DIC just after birth in the current study.

The clinical management of neonatal DIC is controversial. Despite the lack of consensus guidelines for treating neonatal DIC, FFP, antithrombin, and rTM are used. Before these interventions, neonatologists should treat the underlying conditions. Although no pertinent standards have been established, FFP transfusions to neonates should be considered in the clinical context of bleeding, coagulation factors consumption by DIC, and very rare inherited deficiencies of coagulation factors [22, 23]. However, FFP should not be used as a volume expander or for prophylaxis against IVH [24]. In adults, rTM and antithrombin were reported to be effective for treating sepsis-associated DIC [2, 25]. Recently, Shirahata et al. insist that rTM is effective for neonatal DIC on the fact of a high rate, 47%, DIC resolution rate on the day after the last administration of rTM, and a high survival rate, 76% at 28 days after the last administration, although not only neonates diagnosed at birth, but also neonates subsequently diagnosed were included [11]. On the other hand, in the present study we found that the DIC resolution rate one day after rTM administration was almost 70%. This difference in DIC resolution rate between the two studies may be attributed to having different cohorts and treatments. The present study focused on neonates at birth, mainly neonates with DIC associated with birth asphyxia, but only one case of sepsis-associated DIC, whereas Shirahata et al. investigated neonates from the first day of life to Day 28. Moreover, we treated not only with rTM but also with a combination of rTM and FFP. JSOGNH guidelines for diagnosis and clinical management of neonatal DIC published in 2016 recommend that rTM and FFP should be used for neonatal DIC if the neonates have bleeding symptoms, a PT-INR of more than 2.0, an aPTT of less than 25%, or an FBG of less than 100 mg/dl [7]. To date, there have been few reports that validate the effectiveness of FFP for neonatal DIC using DIC scores and DIC resolution in neonates at birth.

Using FFP as a treatment for DIC, we observed that DIC scores increased two days after the first FFP administration, with the increase on Day 2 significantly higher than on Day 1. In contrast, DIC scores and clinical resolution showed gradual, steady improvement in the rTM treatment group. These findings suggest that rTM or the combination of rTM and FFP were effective for neonatal DIC just after birth. Although there are few reports showing the efficacy of early DIC treatment, a previous retrospective study suggested that the efficacy of treatment in relation to the DIC score when the treatment began showed that greater efficacy was achieved in pre-DIC than in DIC adult patients [26]. It is considered that neonates with an underlying condition at birth, such as birth asphyxia, should receive a coagulation workup to see whether DIC treatment is needed.

Our study has several limitations. First, we could not assess the effects of rTM and FFP treatment on sepsis-associated DIC in neonates [5, 25]. Second, neonatal coagulation parameters were collected retrospectively, and protein C levels or other markers, such as soluble fibrin monomer complex were not evaluated. Veldman et al. suggested that platelet consumption and reduced protein C plasma levels could be of diagnostic value in neonatal DIC. However, avoiding phlebotomy precludes investigation of protein C in DIC neonates [4]. On the other hand, Selim et al. suggested that soluble fibrin monomer complex would be a useful biomarker in DIC with neonatal sepsis [27]. Our third limitation is that we used several treatments for DIC. Since our previous study reported that AT levels in premature infants were almost 28% [8], we often used AT supplementation for neonatal DIC along with rTM and FFP treatments. Many studies reported the efficacy of AT supplementation in adult patients with sepsis-associated DIC [28, 29]. It is considered that AT supplementation would be effective for neonatal DIC. Finally, in this study, we used newly proposed neonatal DIC diagnostic criteria from JSOGNH. Of 55 neonates treated as DIC, within our treatment cohort, 22 neonates had suspected DIC. We treated these babies with lower DIC score (DIC scores = 3). The guideline recommended that early intervention of neonatal DIC was needed. However, on this basis, it is very difficult to decide to treat the babies with a borderline DIC score (DIC scores = 3). Decision thresholds vary among NICUs. Investigation into the accuracy of the criteria of JSOGNH is necessary, as is the evaluation of whether these neonates with underlying conditions, such as birth asphyxia, need DIC treatment. Therefore, further extensive, multicenter, prospective studies are warranted to place the findings of this study in a broader clinical context.

In conclusion, the present study demonstrates that various factors are associated with neonatal DIC at birth, with birth asphyxia among the most significant of these factors. Furthermore, rTM in combination with FFP therapy was effective for neonatal DIC at birth.

## Supplementary information

**Additional file 1.** Supplementary Table 1. Coagulation parameters and characteristics of patients with and without underlying disease.

**Additional file 2.** Supplemental Digital content - Table 2. Details of underlying conditions.

**Additional file 3.** Supplementary Digital content-Table 3. Details of DIC treatment among 55 treated neonates.

## Data Availability

All data is available.

## Abbreviation

DIC: disseminated intravascular coagulation
FFP: fresh frozen plasma
PA: placental abruption
PIH: pregnancy induced hypertension
rTM: recombinant thrombomodulin
SGA: small for gestational age
