# Supplementary material for: Risk factors and treatments for disseminated intravascular coagulation in neonates
Source: Ital J Pediatr. 2020 Apr 29;46:54. doi: 10.1186/s13052-020-0815-7 (PMC7191786; doi:10.1186/s13052-020-0815-7)
Supplement: Supplementary file 1 — Additional file 1. Supplementary Table 1. Coagulation parameters and characteristics of patients with and without underlying disease. [file 13052_2020_815_MOESM1_ESM.doc]

Supplementary Table 1. Coagulation parameters and characteristics of patients with and without underlying disease

|  | Underlying condition (n=366) | No underlying condition (n=243) | P-value |
| --- | --- | --- | --- |
| GA (w) (median) | 28.5 | 37.0 | ＜0.05 |
| BW (g) (median) | 1,188 | 2,507 | ＜0.05 |
| PLT (×10^3^/ul) (median) | 220 | 241 | ＜0.05 |
| PT-INR (median) | 1.31 | 1.20 | ＜0.01 |
| APTT (seconds) (median) | 63.7 | 54.8 | ＜0.01 |
| FBG (mg/dl) (median) | 124 | 147 | ＜0.01 |
| D-dimer (ng/ml) (median) | 5.2 | 3.0 | ＜0.01 |
| AT activity (%) (median) | 29 | 42 | ＜0.01 |
| DIC score (median) | 2 | 1 | ＜0.01 |
| IVH (%) (median) | 41（11%） | 7(2%) | ＜0.01 |
| Apgar Score (1min) (median) | 4 | 8 | ＜0.01 |
| Apgar Score (5min) (median) | 6 | 9 | ＜0.01 |

Continuous variables were presented as medians. Mann-Whitney U test, χ^2^ test
