# Supplementary material for: Risk factors and treatments for disseminated intravascular coagulation in neonates
Source: Ital J Pediatr. 2020 Apr 29;46:54. doi: 10.1186/s13052-020-0815-7 (PMC7191786; doi:10.1186/s13052-020-0815-7)
Supplement: Supplementary file 2 — Additional file 2. Supplemental Digital content - Table 2. Details of underlying conditions. [file 13052_2020_815_MOESM2_ESM.doc]

Supplemental Digital content - Table 2. Details of underlying conditions

| BW | ＜1,500g | ≥ 1,500g | P-Value |
| --- | --- | --- | --- |
| n | 168 | 198 |  |
| GA | 26.8 | 36.4 | P<0.001 |
| IVH after birth | 26 | 3 | P<0.001 |
| IVH at birth | 11 | 1 | P=0.001 |
| Apgar Score (1 min) | 4 | 4 | NS |
| Apgar Score (5 min) | 7 | 7 | NS |
| Sepsis | 1 | 0 | NS |
| Birth Asphyxia | 154 | 155 | P<0.001 |
| RDS | 65 | 56 | P=0.03 |
| Hemangioma | 0 | 2 | NS |
| Hydrops | 0 | 9 | P=0.005 |
| PIH | 16 | 29 | NS |
| PA | 16 | 16 | NS |
| Vanishing twin | 2 | 1 | NS |
| DIC treatment | 37 | 18 | P<0.001 |

GA, gestational age; IVH, intraventricular hemorrhage; BW, birth weight; PT-INR, prothrombin time international ratio; RDS, respiratory distress syndrome; PIH, pregnancy induced hypertension; PA, placental abruption; DIC, disseminated intravascular coagulation; NS, not significant

GA, gestational age; IVH, intraventricular hemorrhage; BW, birth weight; PT-INR, prothrombin time international ratio; RDS, respiratory distress syndrome; PIH, pregnancy induced hypertension; PA, placental abruption; DIC, disseminated intravascular coagulation, NS: not significant.
