# Supplementary material for: Risk factors and treatments for disseminated intravascular coagulation in neonates
Source: Ital J Pediatr. 2020 Apr 29;46:54. doi: 10.1186/s13052-020-0815-7 (PMC7191786; doi:10.1186/s13052-020-0815-7)
Supplement: Supplementary file 3 — Additional file 3. Supplementary Digital content-Table 3. Details of DIC treatment among 55 treated neonates. [file 13052_2020_815_MOESM3_ESM.doc]

Supplementary Digital content-Table 3. Details of DIC treatment among 55 treated neonates

|  | N (%) |
| --- | --- |
|  | 55 |
| FFP | 13 (24%) |
| FFP+ATⅢ | 28 (51%) |
| Platelet transfusion | 17 (31%) |
| Blood Exchange | 2 (4%) |
| rTM | 4 (7%) |
| rTM+FFP+ATⅢ | 10 (18%) |

FFP, fresh frozen plasma; rTM, recombinant human soluble thrombomodulin ; ATⅢ, anthrombin Ⅲ
